# Notes and News

**Published:** 1887-04-02

**Authors:** 


### Notes and News.

COLLECTED AND COMMUNICATED.

SURGEON-GENERAL WILLIAM RUTHERFORD, M.D., C.B., Honorary Physician to the Queen, died on the 24th ult., in his 71st year.

IT has been found desirable to go to press later in the week. In consequence of this new arrangement subscribers will not, for the future, receive their copies until Saturday morning. THE HOSPITAL is not due until Saturdays, and as the extra time thus secured is found to be desirable in many ways, it is hoped the change will in no way inconvenience our readers and subscribers.

SPEAKING at the festival dinner in aid of the Victoria Hospital for Children, held on March 29th, 1882, the Prince of Wales remarked, "Thirty years ago there was no hospital for children at all, and though there are many now, I feel sure that the Victoria Hospital, which is situated in the thickly populated district of Chelsea, is one of the most necessary and important."

THE class of Royal Engineer officers held by the St. John Ambulance Association at Chatham, and instructed by Surgeon-Major McNalty, has been examined by the principal medical officer of the district, Deputy Surgeon General E. Roberts, and, out of thirty-four candidates, the whole are reported to have passed an excellent examination in first aid to the injured.

A GOOD dentifrice is a thing, when found, to be made a note of. We therefore make no apology for reproducing one from *The Lancet*. Precipitated chalk forms the best basis for a tooth-powder, to the base of which may be added pulv. saponis and ol. eucalypt., a drachm of each ; and if there is no objection to the taste, half a drachm of carbolic acid.

ON the 26th ult. a drawing-room meeting was held, by the kindness of Mr. and Mrs. Brindly Nixon, at 27, Collingham Gardens, South Kensington, in aid of the East London Nursing Society, the Bishop of Bedford presiding. The Society now employs twenty nurses, who are constantly at work in the homes of the sick poor of the East of London. A lady has generously undertaken to defray the cost of an additional nurse, who will shortly be added to the Society's staff.

THE sick poor—at least at some of our Poor-law Infirmaries—are not to be forgotten during Jubilee week. Special diet, entertainments, etc., are already the subject of consideration at some of our Boards of Guardians. Both the inmates and the officers of the Bow Infirmary, the workhouses at Homerton, and at Shoe Lane are to celebrate the Jubilee by a dinner.

THE Earl of Strafford thinks that the best way of testing the efficiency of the work of a hospital is to visit it unawares. His lordship, who presided at the annual meeting of the subscribers to the National Dental Hospital in Great Portland Street, stated that some time since he paid an *incognito* visit to the institution. He made a thorough inspection of the work, and confessed himself struck with the great kindness and attention shown to the patients, and the admirable way in which everything appeared to be conducted.

IN these days of higher civilisation and luxury it is well to be reminded of the rough and ready country experience of our brethren beyond seas. Dr. L. Howe of Wellston, Ohio, has recently recorded a few of his own, which are, to say the least, a little unusual. He writes :—

"When I entered practice it was in the hills of West Virginia. Was called, with another physician, to amputate an arm, patient living nine miles away. It turned out neither of us had more instruments than a small curved bistoury and a scalpel, with some needles and silk. Not to be balked (as we were much in need of the fee), we borrowed a pocket-knife of a friendly machinist, wrapped the handle with newspaper, and went on rejoicing. My friend administered an anæsthetic while I amputated, three inches below the elbow, an arm as large as an ordinary man's calf. We now discovered that we had forgotten to bring a bone-saw, which discovery had been pre-arranged. Called for and got a rusty hand-saw, which rattled over the bones until the sweat poured down from the face of the operator like rain. Patient made a rapid recovery, which fact alone saved us from the wrath of 'regulators,' who threatened us, should our patient die, with a hemp cord."

Read and ponder this, you sentimentalists who growl at the thought of waiting for a couple of hours or so in the out-patient room of a busy London hospital.

AT the present time, when legacies to hospitals are decreasing in amount and frequency, it is pleasant to be able to report the action of a gentleman who knew much about hospitals, and who always stood their friend. The late Dr. Wakley, editor of *The Lancet*, has bequeathed to University College Hospital his freehold residence, Heathlands, Longcross, Chertsey, and eight acres of land, for the purposes of a convalescent home for patients from this hospital, as a memorial of his late father, Thomas Wakley, Esq., M.P., the founder of *The Lancet*. The home is to be called the "Wakley Convalescent Home." Dr. Wakley also bequeathed £1,000 to be expended in the maintenance of the convalescent home, at the rate of £200 per annum, and another £1,000 to the Metropolitan Hospital Sunday Fund. May many be found to follow the example of one who had the deepest sympathy with suffering and the sick.

The following vacancies are announced this week, the amount of the salary being placed in each case after the name of the institution :—

**Honorary Medical Officer.**

Farringdon Dispensary, Holborn. Physician.

**Resident Medical Officers.**

Bradford Infirmary. House Surgeon, doubly-qualified.—£110.
Brompton Consumption Hospital. Clinical Assistant.
Barming Heath Asylum, Kent. Third Assistant.—£120.
Bristol Hospital for Sick Children. House Surgeon, doubly-qualified.—£120.
Gloucester County Asylum. Third Assistant, fully-qualified. —£105.
London Temperance Hospital, N.W. Senior House Surgeon, doubly-qualified.—£52 10s.
Royal General Dispensary, E.C. One, doubly-qualified.—£130.

**Non-resident Medical Officer.**

University College. Professor of Chemistry.

**Matrons.**

Crewkerne Cottage Hospital.—£30.
Hospital for Epilepsy, Regent's Park.—£50.

**Trained Nurses.**

Coventry Nursing Association. One.—£30.
Hampstead Home Hospital. Several.—£20.
New Hospital for Women, Marylebone Road. Assistant Nurse. —£18.
Pontefract Union Infirmary. One.—£25
Queen Charlotte's Hospital. Two midwives.—£40.
St. Albans Hospital. One.—£35.

**Probationer.**

Newbury District Hospital. One.—£12.

An urgent appeal has reached us on behalf of the enlargement fund of Northampton General Infirmary, and under the peculiar circumstances of the case we think it well to give our charitable readers an opportunity of helping a deserving institution to meet the calls made upon it. The town of Northampton has greatly increased since the present buildings were erected, being more than four times its original size, and of course the population in the surrounding district has not stood still. In consequence of this state of things patients have frequently to be sent back to their homes unrelieved, and of late it has been necessary to reject as many as fifteen to eighteen sufferers on several consecutive Saturdays, whilst others have to be discharged sooner than would be considered desirable if it were not that their room was urgently required for fresh patients. A committee appointed in 1885 recommended the building of a wing, to include a children's ward, a convalescent ward, and some additional rooms for the accommodation of the nursing staff. To accomplish this work it is estimated that nearly £10,000 will be required, and it is with the object of raising this sum that the present appeal has been issued. Money is not too plentiful in these hard times, but we greatly hope that the committee of Northampton Infirmary may be able to raise the necessary funds for their proposed improvements.

The *New York Medical Record* has the following weighty utterance on a matter of great public importance. We commend these words of warning to women, their husbands and friends, who will do well to take Dr. Duncan's belief to heart, and to weigh his views and to act upon them. In this age of specialism it is desirable to remember that specialists must live, and to live they must have patients. Hence Dr. Duncan's public-spirited utterances are the more valuable, because wholly disinterested, and to the public advantage :—"Mr. Tait, writing to the *British Medical Journal* regarding the Liverpool Hospital quarrel, says of Dr. Duncan, what we believe is more of a compliment than he intends. He says of him : 'Dr. Duncan's views are warped by a belief which I respect as honest, but regard as wholly mistaken and most unfortunate for "good gynecology." This belief is to the effect that the majority of women who give a detailed, sequent, and consistent story of pelvic suffering, "have nothing the matter with them." This has passed into a byword, and has immensely diminished Dr. Duncan's sphere of usefulness in the practice of medicine.' We venture to say that Dr. Duncan holds no such extreme view as represented, and that if gynecologists with pet operations dominating their minds were to absorb a little of Dr. Duncan's conservatism their sphere of usefulness would be greatly enlarged."

The deep interest which the Duke of Cambridge takes in our charitable institutions was well evidenced by his speech at the fortieth anniversary festival in aid of the Earlswood Asylum for Idiots, held last week at Willis's Rooms. The well-known asylum at Redhill, which was first established at Highgate in 1847, may well claim the title of a national institution, for its inmates are gathered from every quarter of the United Kingdom. The idiot is, as His Royal Highness remarked, an absolute clog and trouble to his poor family. When one remembers that the distressful mental malady is probably for life, it is clear how great is the boon of such an institution as the Earlswood Asylum. Cure is, of course, not to be hoped for, but the resources of science can mitigate the suffering of the idiot. At the present moment there are 564 inmates housed at Earlswood, and during the present year fifty more are to be admitted. The accommodation at the asylum is sufficient for 650, but the limited funds of the institution demand the exercise of the utmost caution in filling up vacancies. Mr. William Nichols, who has acted as secretary to the asylum for the lengthened period of thirty-eight years, is about to relinquish his post.

We have no sympathy with quacks and pretenders to medical knowledge, but the case of Lemonnier, described in the following paragraph, which we extract from the *Evening Standard* of the 25th of March, seems rather a hard one, supposing the testimony in his favour to be genuine.

"A mock doctor, named Lemonnier, has been condemned to five years' solitary confinement and transportation for the illegal practice of medicine. He had succeeded in making a lot of money by using a forged certificate of his qualifications as a medical man, and led a jovial life in Toulon and Paris until he was found out. He had been in Toulon during the cholera epidemic which raged about two years ago in that city, and had rendered valuable services in the hospitals and houses of the stricken. He would even have received a commemorative medal or decoration for his devotedness in the cause of the sick had he not been denounced as a rogue. As it was, his exemplary conduct during the cholera epidemic had been observed by a high naval official, who was about to procure him a commission as a surgeon in the fleet. Having been unmasked in Toulon, Lemonnier fled to Brittany, and was arrested in Morbihan. When brought up for trial he shammed dumbness, and answered all questions by writing on a slate."

THE following sums have been recently received by the various Medical Charities, the name of the donor or the source from which they were obtained being given in each instance after the name of the Institution :—

**Donations.**

Cancer Hospital, Brompton. F., 10 guineas.
City of London Lying-in Hospital, City Road, E.C. J. de la Rue and Co., £10 10*s*. M. Brass and Son, £10 10*s*.
Dental Hospital of London. Mr. James Mowatt, 10 guineas. Anon., 30 guineas W. G., £25. Mr. Daniel Browning, L.D.S. Eng., 12 guineas.
King's College Hospital. W. L. T. R., £20.
North London Hospital. Mr. F. D. Mocatta, 10 guineas.
Queen Charlotte's Lying-in Hospital, Marylebone Road. Mr. F. H. Burrows, 20 guineas.
Royal Caledonian Asylum, Holloway. Sir Geo. Stephen, Bart., 100 guineas.
Royal Hospital for Children and Women, Waterloo Bridge Road. Mr. Bertram Barton, 25 guineas. Mr. Chas. Churchill, 20 guineas (for Special Fund for liquidating debt).

**Entertainments.**

Royal Ear Hospital. Jubilee Concert given by the employés of the Railway Clearing House. 50 guineas.
Victoria Hospital for Children, Chelsea. Concert given by the Children's Orchestra at the Grosvenor Hotel, 28th Feb., £41 10*s*. 6*d*. Entertainment given by the Minstrel Troupe of the Royal Horse Guards at the Chelsea Town Hall, 28th Feb., £5.

**Collections.**

Central London Throat and Ear Hospital, Gray's Inn Road. Miss Dora Jackson's tenth annual collection among her family and friends, £26 2*s*.

---

ACCORDING to the *Indiana Medical Jonrnal*, the professor of surgical anatomy in a physio-medical college in Indianapolis announces in the daily papers that he possesses a remedy which " will cure membranous croup every time, and is the only thing that will." The formula is as follows :

| | |
|---|---|
| Tr. sanguinaria .. .. .. | .. drachms iv. |
| Fl. ex. spikenard .. .. .. | .. drachms ij. |
| Oil pennyroyal .. .. .. | .. gtt. ij. |
| Simp. syr. (heavy) .. .. | q. s. ad ounces iv. |

Mix.—Dose for a child of one year, ten drops, and add five more for each additional year. Give every half-hour, hour, or two hours, as the urgency may require. What would such a line of conduct be thought of in this country to-day ?

---

THE special fund now in course of collection to lift Guy's out of its difficulties, is not progressing very fast. It amounts to a little over £53,000, or rather more than half the amount asked for. To this must be added Mr. J. S. Morgan's princely donation of £10,000, given conditionally on the balance being forthcoming by May 1st—now only a month off. " A friend" has, through Mr. Weylland, offered £500 to follow Mr. Morgan's donation, and Sir Henry Peak, in addition to a gift of £500 already made, has promised, upon like terms, a further contribution of £250. A generous donor, who wishes to be known as J. G., has given £500 to Guy's Special Fund.

---

ALDERMAN GOLDSCHMIDT presided recently at a meeting of the Board of Management of the Manchester Royal Infirmary, when Mr. O'Hanlon explained the proposal of the Monsall Hospital Committee to build another pavilion, resembling the one last erected, the cost of which came to about £3,000. An epidemic of scarlet fever which has lately prevailed in the district has been the chief factor in the very large increase, about 260 per cent., in the number of patients treated at Monsall.

THE Princess of Wales, with the three young Princesses, paid a private visit to the Brompton Hospital on Friday afternoon last. Their Royal Highnesses took part in an entertainment given to the patients and nurses in the Concert Room, and afterwards visited the "Alexandra" Gallery, and the two men's floors above. The Princess, with charming grace, and words of the kindest sympathy, distributed individually the flowers which she had so thoughtfully brought with her for that purpose.

---

THE lock ward of the Royal Albert Hospital at Devonport is to be re-opened on the 1st April, this most needful step having been rendered possible by the decision of the Admiralty to pay the Hospital Committee £35 for each of thirty beds in the ward. We are glad to know that the Admiralty Board is doing its best to remedy the terrible evil likely to ensue from the rash and misguided action of Parliament.

---

THE Corporation of Manchester have decided to sell the Wilton Hospital for infectious diseases to the London and North Western Railway Company for the sum of £21,000, subject to the sanction and approval of the Local Government Board. This has been rendered necessary by the construction of sidings for a cattle station on land fronting and abutting on the hospital, which was certain to occasion extreme annoyance and inconvenience to the patients and authorities of the hospital. Councillor Middlehurst, speaking at the meeting of the Corporation to decide this question, said that the Wilton Hospital had been regarded as a white elephant on their hands, and begged to congratulate the Committee on so advantageous a sale of the property.

---

THE building of the new convalescent home at Cookridge, in connection with the Leeds Infirmary, will, we believe, be begun very shortly, as the plans have been decided upon. The accepted design, one of three sent in by Messrs. Chorley and Connon, shows a central administrative block with wings only one storey high, containing wards for the accommodation of forty-two patients, viz., two wards of sixteen beds each, two of four beds each, and two single wards. The design provides for a dispensary, consulting-room, clothing stores, medical officer's room, kitchen, scullery, and other offices on the ground-floor of the central build ing, whilst on the first-floor and in the attics there are rooms for the matron and the nursing staff. Steam pipes for heating the wards, corridors, etc., are provided throughout, and the necessary ventilation will be performed by the aid of Harding's diffusers. The outside will be faced with red brick, having a stone base and dressing, the upper portion of the central building being executed in half timber and rough-cast, whilst the roofs are to be covered with green slates. The cost of erection has been undertaken by Mr. John North, who has lately increased his original gift of £4,000 to the building fund, to £5,000, and has even promised more if it should be found necessary. The committee are, however, in hopes of obtaining a surplus from Mr North's gift to be expended on furnishing.

